# Effects of cardiac resynchronization therapy in adults with congenital heart disease

**DOI:** 10.1186/s12872-026-05818-5

**Published:** 2026-04-07

**Authors:** Ralph M.L. Neijenhuis, Bert A.C. Zwaenepoel, Thomas D. Jansen, Vivienne Ezzat, Lieselot van Erven, Martin Lowe, Martin J. Schalij, Vinit Sawhney, Saskia L.M.A. Beeres, Holly L. Daw, J. Wouter Jukema, Monique R.M. Jongbloed, Filip Zemrak, Anastasia D. Egorova

**Affiliations:** 1 CAHAL, Center for Congenital Heart Disease Amsterdam-Leiden, Leiden University Medical Center, Leiden, The Netherlands; 2 Department of Cardiology, Leiden University Medical Center, Leiden, The Netherlands; 3 Department of Cardiology, Algemeen Ziekenhuis Delta, Roeselare, Belgium; 4 Barts Heart Centre, St. Bartholomew’s Hospital, Barts Health NHS Trust, London, UK; 5 William Harvey Research Institute, Barts and The London School of Medicine and Dentistry, Queen Mary University of London, Charterhouse Square, London, UK; 6 Netherlands Heart Institute, Utrecht, The Netherlands; 7 Department of Anatomy and Embryology, Leiden University Medical Center, Leiden, The Netherlands; 8 Institute of Cardiovascular Science, University College London, London, UK

**Keywords:** Cardiac Resynchronization Therapy, CRT, biventricular pacing, Adult Congenital Heart Disease, ACHD, heart failure, HF

## Abstract

**Background:**

Cardiac resynchronization therapy (CRT) is well-established in acquired heart failure, but evidence in adults with congenital heart disease (ACHD) remains limited. Current guidelines, extrapolated from non-congenital populations, may not fully address the anatomical and electrophysiological complexities in ACHD. This study aimed to evaluate the safety and efficacy of CRT in ACHD, focusing on changes in QRS duration, systemic ventricular function (SVF), and New York Heart Association (NYHA) class.

**Methods:**

A retrospective multicenter cohort study was conducted across two tertiary ACHD centers. ACHD patients who underwent CRT implantation between 2014 and 2024 were included. Primary outcomes were QRS duration, SVF, and NYHA class at 3, 6, and 12 months post-implantation, and peri-procedural clinical and device-related outcomes were assessed. Mixed models were used to analyze longitudinal changes, adjusting for de novo vs. upgrade CRT implantation, left bundle branch block (LBBB) vs. non-LBBB QRS morphology, and systemic left vs. right ventricle (sRV) anatomy.

**Results:**

101 patients were included. The mean age was 53 ± 14 year, 41 (40.6%) were female and 48 (47.5%) underwent an upgrade procedure to CRT. Twenty-two patients (21.8%) had mild, 57 (56.4%) moderate, and 22 (21.8%) severe congenital heart disease, and 16 (15.8%) had a sRV. Six patients (5.9%) experienced a procedure-related complication requiring revision (6.6 per 100 patient-years follow-up), and one patient (1.0%) died from end-stage heart failure in the year after CRT implantation. CRT was associated with significant improvements in QRS duration, SVF, and NYHA class at 3, 6, and 12 months (*p* < 0.05 for all). QRS reduction was more pronounced after upgrade procedures. Improvements in QRS, SVF, and NYHA class extended to patients with a sRV and with non-LBBB QRS morphology. At one year follow-up, 68 patients (72.3%) showed improvement of at least 1 class of NYHA and/or SVF.

**Conclusions:**

This study supports CRT as a safe and effective therapy in ACHD patients, including those with sRV or non-LBBB QRS morphology, emphasizing the need for individualized decision-making beyond standard guidelines.

**Graphical Abstract:**

**Figure:**
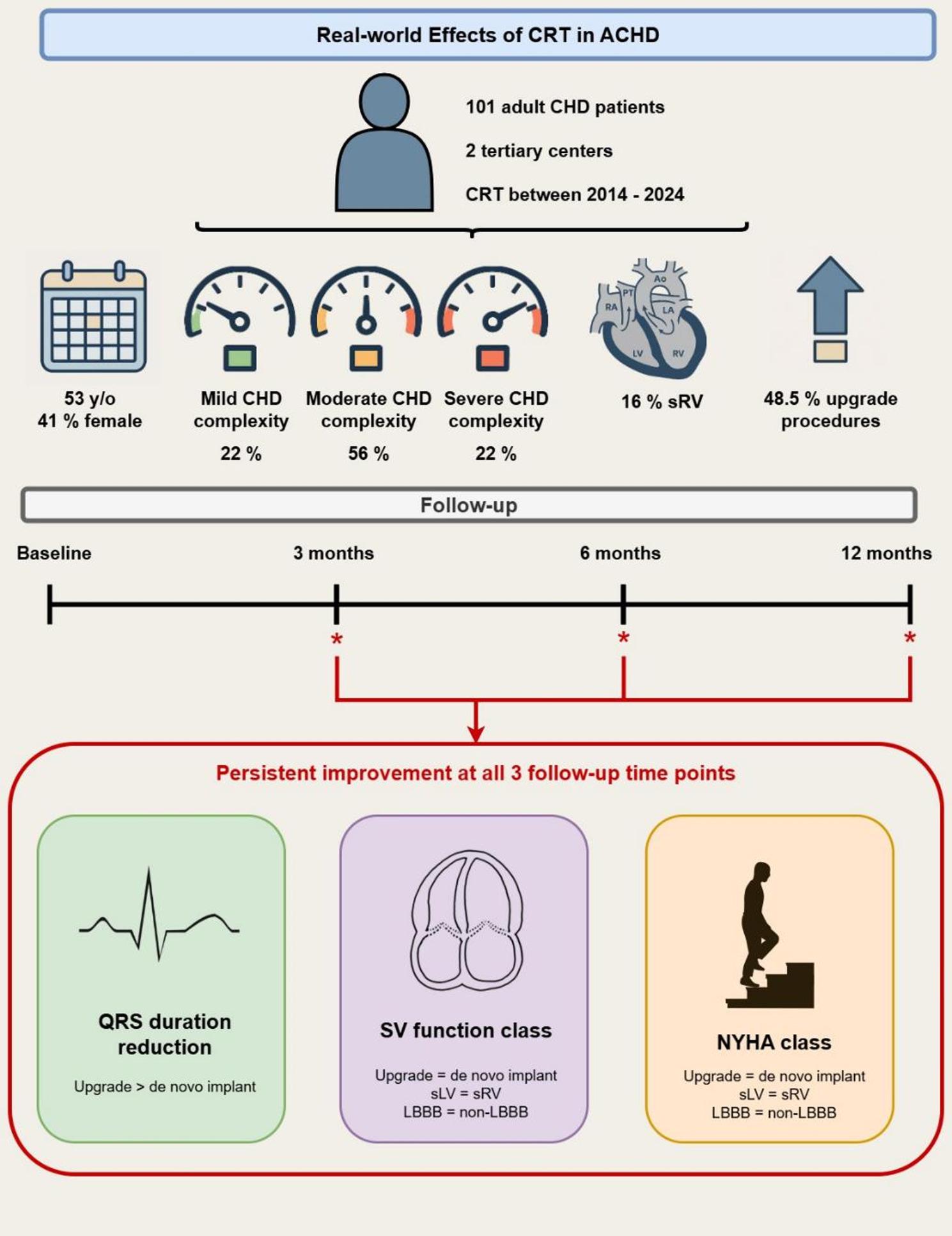
Effects of CRT in ACHD. In this real-world retrospective multicenter cohort study, the effects of CRT were investigated in 101 ACHD patients. CRT was associated with significant improvements in QRS duration, SVF, and NYHA class at 3, 6, and 12 months after implantation (*p* < 0.05 for all). While QRS reduction was more pronounced after upgrade procedures than in de novo implants, both groups showed significant improvements in SVF and NYHA class. Moreover, improvements in QRS, SVF, and NYHA class extended to sRV and non-LBBB patients, supporting CRT as a safe and effective therapy in ACHD patients and emphasizing the need for individualized decision-making beyond standard guidelines. *(A)CHD* (adult) congenital heart disease, CRT Cardiac resynchronization therapy, LBBB Left bundle branch block, *NYHA* New York Heart Association, *sLV* Systemic left ventricle, *sRV* Systemic right ventricle, *SVF* Systemic ventricular function.

**Supplementary Information:**

The online version contains supplementary material available at 10.1186/s12872-026-05818-5.

## Introduction

Over the past decades, the clinical practice of congenital heart disease has shifted from childhood mortality towards chronic morbidity in adulthood. As a result, the adult congenital heart disease (ACHD) population is increasing, with more long-term complications, of which heart failure (HF) is now the leading cause of morbidity and mortality [1]. Despite this growing clinical burden, evidence for management strategies of ACHD-related HF is still limited. Therefore, current recommendations are largely extrapolated from studies in HF patients with acquired heart disease [1].

Cardiac resynchronization therapy (CRT) has been demonstrated to be effective in the treatment of electromechanical dyssynchrony in symptomatic HF patients without congenital heart disease, leading to functional improvement and a reduction in HF-associated morbidity and mortality [2–4]. For patients with ACHD with a systemic left ventricle (sLV), current guidelines recommend applying conventional CRT indications [1, 5, 6]. However, these are based on studies in patients with anatomically normal hearts. In ACHD, complex anatomies, surgically induced scar tissue, and associated lesions may influence CRT response [5]. Ill-defined indication criteria and doubts about efficacy of CRT in ACHD may lead to underuse, potentially resulting in suboptimal care for patients [7].

Smaller studies have shown promising results for CRT in ACHD patients, although those patients have often not met the classical guideline-based criteria for CRT [8–11]. Moreover, heterogeneity in patient selection and the lack of a uniform definition of response complicate the interpretation of efficacy and limit comparability between studies [12]. To expand upon previous findings and contribute to closing the existing evidence gap, this multicenter study evaluates real-world data from the largest available cohort of ACHD patients treated with CRT to date. Specifically, the objectives of the study are to assess: (1) the anatomical and clinical characteristics of ACHD patients undergoing CRT implantation (2), the peri-procedural clinical and device-related outcomes, and (3) the longitudinal changes in QRS duration, systemic ventricular function (SVF), and New York Heart Association (NYHA) functional class in the year after CRT implantation.

## Methods

### Study design and population

This retrospective multicenter cohort study included all consecutive ACHD patients who underwent CRT implantation between January 2014 and December 2024 in two European tertiary ACHD centers (Leiden University Medical Center (LUMC), Leiden, the Netherlands, and Barts Heart Centre, St Bartholomew’s Hospital, London, United Kingdom). Both de novo implantations and upgrade procedures to CRT were included. Exclusion criteria were age < 18 years at the time of CRT implantation and patient objection to the use of retrospective data. The study was approved by the ethics committee of the LUMC (Medical Research Involving Human Subjects Act [WMO] committee division 1, protocol reference 2023-035). At Barts Heart Centre, the study was approved in accordance with local institutional policy. All tests and procedures were performed as part of standard clinical care, and the need for written informed consent was waived. The study was conducted in accordance with the ethical standards of the relevant institutional and national research committees and complied with the Declaration of Helsinki.

### Data collection and follow-up

All data were retrieved from the electronic health records at the participating centers. Baseline parameters included patient demographics, past medical history, medication use, clinical parameters at implantation, NYHA functional class, laboratory parameters, 12-lead electrocardiography (ECG), and transthoracic echocardiographic data, where available. Cardiac implantable electronic device (CIED) procedural information was collected, as well as the initial indication for CRT. Follow-up visits were scheduled according to the local post-implantation protocols. For analysis purposes, follow-up assessments were categorized into three predefined time points: 3 (± 1) months, 6 (± 2) months, and 12 (± 4) months post-procedure. Follow-up evaluations conducted within these intervals were categorized accordingly. Follow-up events outside these intervals were excluded. If multiple evaluations occurred within the same interval, the one closest to the target time point was retained. Follow-up data included clinical and ECG parameters, transthoracic echocardiography assessment, CIED/lead parameters, and CIED-related complications. SVF classes were categorized as good, mildly reduced, moderately reduced, or severely reduced, in accordance with the established guidelines [13]. Quantification methods included Simpson’s biplane or three-dimensional ejection fraction, global longitudinal strain, fractional area change (FAC) for systemic right ventricles (sRV), and global visual assessment. Data on HF events (urgent HF visits and HF hospitalizations), referral for left ventricular assist device implantation and/or heart transplantation, and mortality were also collected.

### Statistical analysis

All statistical analyses were performed in SPSS version 25 (IBM Corp, Armonk, NY) and R Statistical Software (v4.4.2; R Core Team 2023). For the descriptive analysis, normally distributed continuous data were presented as mean ± standard deviation, skewed data as median [interquartile range], and categorical data as counts (percentages). Paired Student’s t-tests were used for within-group comparisons of normally distributed continuous variables.

To evaluate longitudinal changes in QRS duration, SVF, and NYHA class at 3, 6, and 12 months following CRT implantation, mixed models were used because they account for the within-patient correlation of repeated measurements and appropriately handle unbalanced data. Mixed models allowed us to include all available data points, providing robust estimates of temporal trends even when not all patients had complete assessments at every time point. The Linear and Nonlinear Mixed Effects Models (nlme) package (v3.1.167; R Core Team 2024) was used to construct linear mixed models for the longitudinal changes in the continuous parameter QRS duration. Cumulative link mixed models were constructed for the ordinal parameter SVF class with the “ordinal” package (v2023.12.4.1; R Core Team 2024). The “lme4” package (v1.1.36; R Core Team 2024) was used to construct generalized linear mixed models for the binary parameter NYHA functional class (NYHA class I or II versus NYHA class > II). Results of the cumulative link mixed models were presented as the cumulative odds ratios for each follow-up time point compared to baseline, with a cumulative odds ratio < 1 indicating an overall improvement in SVF class and a cumulative odds ratio > 1 indicating a worsening. Results of the generalized linear mixed models were presented as odds ratios, with an odds ratio < 1 indicating a lower odds of having NYHA class > II compared to baseline, and an odds ratio > 1 indicating a higher odds of having an NYHA class > II. All data were included in the mixed models, including patients in NYHA class I and those with preserved SVF at baseline, who, by definition, could not demonstrate an improvement by definition but were at risk of deterioration.

The effects of the following three binary covariates were evaluated in each model: (1) de novo versus upgrade CRT procedure, (2) left bundle branch (LBBB) pattern versus non-LBBB pattern on 12-lead ECG at baseline, and (3) sLV versus sRV anatomy. Each covariate was first included with a time interaction to test if the covariate had a significant effect on the outcome of interest after CRT implantation. If this was not significant, the fixed effect was tested without interaction. If this was also not significant, the covariate was not included in the final model. Different correlation and variance structures were tested. Because model parsimony was prioritized over predictive capacity, model selection was primarily based on the Bayesian information criterion (BIC), with secondary consideration of the Akaike information criterion (AIC) or likelihood ratio tests where appropriate. The model fit was evaluated through residual analysis. Fixed effects were visualized with corresponding 95% confidence intervals for fixed-effect variance. A two-sided p-value < 0.05 was considered statistically significant.

## Results

### Baseline characteristics

Overall, 101 ACHD patients underwent CRT implantation between 2014 and 2024. The mean age at implantation was 53 ± 14 years, and 41 (40.6%) were female. All patients had a biventricular circulation, and according to the current European Society of Cardiology classification of congenital heart disease, 22 (21.8%) had mild, 57 (56.4%) moderate, and 22 (21.8%) severe congenital heart disease [14]. The baseline characteristics are summarized in Table 1.

**Table 1:** Patient characteristics at baseline

Baseline characteristics	n=101
Age (y)	53.2 ± 13.6
Body Mass Index (kg/m²) (n=73)	25.8 [21.9 – 28.5]
Sex (female)	41 (40.6%)
Cardiac history	
Mild congenital complexity	22 (21.8%)
Aortic valve disease or BAV	9 (8.9%)
Repaired ASD, Sinus venosus defect, VSD or PDA	13 (12.9%)
Moderate congenital complexity	57 (56.4%)
Repaired TOF	15 (14.9%)
Congenital aortic stenosis (SAS/SVAS)	12 (11.9%)
Aortic coarctation	9 (8.9%)
ACAOS	4 (4.0%)
VSD with associated abnormalities or shunt	4 (4.0%)
AVSD (including primum ASD)	3 (3.0%)
Marfan syndrome (and related HTAD), Turner Syndrome	2 (2.0%)
Pulmonary stenosis	2 (2.0%)
TGA after arterial switch	2 (2.0%)
PAPVR/TAPVR	1 (1.0%)
ASD secundum type unrepaired	1 (1.0%)
Sinus venosus defect unrepaired	1 (1.0%)
Ebstein anomaly	1 (1.0%)
Severe congenital complexity	22 (21.8%)
ccTGA	14 (13.9%)
CHD related to pulmonary vascular disease (incl. Eisenmenger syndrome)	4 (4.0%)
TGA after Mustard atrial switch procedure	2 (2.0%)
Double outlet ventricle	2 (2.0%)
Number of prior cardiac surgeries	1 [1.0 – 2.5]
Systemic left ventricle	85 (84.2%)
Systemic right ventricle	16 (15.8%)
Comorbidities	
Valvular heart disease	33 (32.7%)
Hypertension	26 (25.7%)
Hyperlipidemia	16 (15.8%)
Renal dysfunction (eGFR 30 mL/min/1.73m^2^)	16 (15.8%)
Coronary Artery Disease	14 (13.9%)
Cerebrovascular disease	12 (11.9%)
COPD	6 (5.9%)
Peripheral Artery Disease	4 (4.0%)
Electrocardiographic data (n=98)	
QRS (ms)	179 ± 39
Narrow QRS (< 120 ms)	5 (5.1%)
Broad QRS (≥ 120 ms)	93 (94.9%)
LBBB pattern	33 (33.7%)
RBBB pattern	16 (16.3%)
Ventricular pacing	37 (37.8%)
Ventricular escape rhythm	7 (7.1%)
Types of rhythms	
Pre-CRT subpulmonary ventricular pacing	37 (37.8%)
Sinus rhythm	33 (33.7%)
Pre-CRT atrial pacing	22 (22.4%)
AT/AF or atrial flutter	25 (25.5%)
Ventricular escape rhythm	7 (7.1%)
NYHA functional class (n=94)	
I – II	48 (51.1%)
> II	46 (48.9%)
Echocardiography	
Systemic ventricle ejection fraction biplane (%) (n=65)	31.4 ± 10.6
Systemic ventricle function class (%) (n=96)	
Good	4 (4.2%)
Mildly reduced	13 (13.5%)
Moderately reduced	44 (45.8%)
Severely reduced	35 (36.5%)
SAVV regurgitation class (%) (n=86)	
I	32 (37.2%)
II	32 (37.2%)
III	15 (17.4%)
VI	7 (8.1%)
Heart failure medication	
ACEi/ARNI/ARB	77 (76.2%)
Diuretics	56 (55.4%)
MRA	43 (42.6%)
Beta-blockers	76 (75.2%)
SGLT2i	10 (9.9%)

Proportions are presented as n (%). Values as mean ± standard deviation or median [interquartile range], as appropriate. Data were available for all 101 patients unless otherwise specified

*AAOCA* Anomalous Aortic Origin of a Coronary Artery, *ACAOS* Anomalous Coronary Artery from the Opposite Sinus, *ACEI* Angiotensin-Converting Enzyme Inhibitor, *ARB* Angiotensin Receptor Blocker, *ARNI* Angiotensin Receptor-Neprilysin Inhibitor, *ASD* Atrial Septal Defect, *ASD-I* Atrial Septal Defect Type I, *ASD-II* Atrial Septal Defect Type II, *AT/AF* Atrial tachycardia/atrial fibrillation, *AOS* Aortic Stenosis, *AVSD* Atrioventricular Septal Defect, *BAV* Bicuspid Aortic Valve, *ccTGA* Congenitally Corrected Transposition of the Great Arteries, *COPD* Chronic Obstructive Pulmonary Disease, *CRT* Cardiac Resynchronization Therapy, *TGA* Transposition of the Great Arteries, *DORV* Double Outlet Right Ventricle, *eGFR* Estimated Glomerular Filtration Rate, *HTAD* Heritable Thoracic Aortic Disease, *LBBB* Left Bundle Branch Block, *MRA* Mineralocorticoid Receptor Antagonist, *NYHA* New York Heart Association, *PAPVR* Partial Anomalous Pulmonary Venous Return, *PDA* Patent Ductus Arteriosus, *PFO* Patent Foramen Ovale, *RBBB* Right Bundle Branch Block, *SAS* Subaortic Stenosis, *SAVV* Systemic Atrioventricular Valve, *SGLT2i* Sodium-Glucose Cotransporter 2 Inhibitor, *SVAS* Supravalvular Aortic Stenosis, *TAPVR* Total Anomalous Pulmonary Venous Return, *TOF* Tetralogy of Fallot, *VSD* Ventricular Septal Defect

The most common diagnoses were systemic right ventricle (sRV) in 16 patients (15.8%), including 14 with congenitally corrected transposition of the great arteries (ccTGA, 13.9%) and 2 with transposition of the great arteries (TGA) after a Mustard atrial switch procedure (2.0%), and tetralogy of Fallot (*n* = 15, 14.9%).

At baseline, 33 patients (33.7%) were in sinus rhythm and 25 (25.5%) in atrial tachycardia/atrial fibrillation or flutter. The mean QRS duration on 12-lead surface ECG was 179 ± 39 ms; LBBB pattern was present in 33 patients (33.7%) while right bundle branch block (RBBB) pattern in 16 (16.3%). Thirty-seven patients (37.8%) had subpulmonary ventricular pacing and seven patients (7.1%) had underlying complete heart block with a broad ventricular escape rhythm. Five patients (5.1%) had a narrow QRS complex, which included 3 cases of post-operative complete heart block in whom a CRT was implanted directly in anticipation of a high percentage of ventricular pacing, and 2 cases with atrial fibrillation planned for a pace-and-ablate strategy. Among the 14 patients with ccTGA, 3 exhibited a LBBB pattern on 12-lead surface ECG, 1 had a RBBB pattern, 9 had subpulmonary ventricular pacing, and baseline ECG was unavailable for 1 patient. Of the 2 patients with TGA after atrial switch, one had subpulmonary ventricular pacing at baseline, while the other demonstrated an RBBB pattern on 12-lead surface ECG.

In the complete cohort, SVF was good in 4 (4.2%), mildly reduced in 13 (13.5%), moderately reduced in 44 (45.8%), and severely reduced in 35 patients (36.5%). Forty-six patients (48.9%) were in NYHA functional class > II at CRT implantation.

### Indication for device implantation/upgrade

Device-related data are presented in Table 2. Nearly half of the patients (*n* = 48, 47.5%) had a pre-existing pacing system and underwent an upgrade to CRT. Most patients (*n* = 86, 85.1%) had a CRT indication according to the current guidelines [15]. Fifteen patients (14.9%) received CRT outside of conventional guideline indications. In 6 of them, a high ventricular pacing burden had already led to a decline in SVF to mildly reduced levels, prompting the expert team to proceed with CRT without awaiting further deterioration. In 8 patients, a high anticipated pacing need combined with mildly reduced SVF was considered sufficient rationale to implant CRT proactively; 3 of these 8 patients still had preserved SVF at the time of implantation. One additional patient underwent CRT-D implantation for secondary prevention of sudden cardiac death in the setting of RBBB and mildly reduced SVF. No patients received conduction system pacing (either His bundle pacing or left bundle branch area pacing).

**Table 2:** Device-related data

Technical CRT data	n=101
CRT-P	37 (36.6%)
CRT-D	64 (63.4%)
CIED pre-CRT	48 (47.5%)
Systemic ventricle lead location	
Posterolateral	38 (37.6%)
Lateral	31 (30.7%)
Anterolateral	17 (16.8%)
Epicardial	11 (10.9%)
Posterior	3 (3.0%)
Inferolateral	1 (1.0%)
Systemic ventricle lead polarity	
Quadripolar	64 (63.4%)
Bipolar	26 (25.7%)
Unipolar	11 (10.9%)
Total leads post-CRT	
Three leads	69 (68.3%)
Four leads	17 (16.8%)
Two leads	9 (8.9%)
Five leads	6 (5.9%)
Systemic ventricle lead impedance (Ω, n=85)	735 [592 – 1005]
Systemic ventricle lead capture threshold (J/s, n=91)	1.1 [0.8 – 1.6]
Systemic ventricle lead impulse width (ms, n=89)	0.4 [0.4 – 0.5]
Systemic ventricle lead sense (mV, n=43)	7.5 [1.9 – 17.4]
Reason for CRT implantation	
Conventional class I – IIb indication *	86 (85.1%)
Indication outside established guidelines *	15 (14.9%)

Proportions are presented as n (%). Values median [interquartile range]. Data were available for all 101 patients unless otherwise specified. * Guidelines refer to the 2021 ESC Guidelines for the diagnosis and treatment of acute and chronic heart failure. *CIED* Cardiac Implantable Electronic Device, *CRT* Cardiac Resynchronization Therapy, *CRT-D* Cardiac Resynchronization Therapy with Defibrillator, *CRT-P* Cardiac Resynchronization Therapy with Pacemaker, *LBBB* Left Bundle Branch Block, *LVEF* Left Ventricular Ejection Fraction

### Periprocedural course and device-related follow-up

Of 101 patients, 37 (36.6%) received a CRT-pacemaker and 64 patients (63.4%) a CRT-defibrillator. The implantation approach was transvenous in 90 (89.1%) of patients, while the remaining 11 patients underwent surgical epicardial lead placement. Lead positioning was guided by coronary venous anatomy, inter-lead spacing, lead stability, and electrical parameters. The transvenous systemic ventricular lead was implanted in the lateral (*n* = 31, 34.4%) or posterolateral (*n* = 38, 42.2%) region in the majority of cases.

At one year follow-up, 11 patients (10.9%) had 14 CRT-related early complications (15.3 per 100 patient-years follow-up), with 6 (5.9%) requiring pocket and/or lead revision (6.6 per 100 patient-years follow-up) (Table 3). Of these complications, lead displacement (*n* = 5) and pocket hematoma (*n* = 4) were the most prevalent. Only one patient had an infection, which was limited to the skin and subcutaneous tissue, without communication with the pocket and thus did not require lead or device extraction. The infection resolved with a course of antibiotics (flucloxacillin, in consultation with the microbiology department). In total, 3 ICD shocks were delivered, of which 2 were appropriate. However, it is important to note that these incidence rates reflect only early complications (i.e., within the first year after CRT).

**Table 3:** Follow-up data

Procedure-related complications	*n* = 14
Major complications (required revision procedure)	6 (5.9%)
Lead displacement	5 (5.0%)
Reversed A and V leads in header	1 (1.0%)
Minor complications (managed with conservative treatment)	8 (7.9%)
Hematoma	4 (4.0%)
Superficial incisional infection	1 (1.0%)
Self-containing dissection of superior vena cava	1 (1.0%)
Bleeding	1 (1.0%)
Deep venous thrombosis	1 (1.0%)
Heart failure related events	*n* = 10
Urgent heart failure visit	8 (80.0%)
Heart failure hospitalization	2 (20.0%)
ICD therapy	*n* = 3
Appropriate ICD shocks given	2 (66.6%)
Inappropriate ICD shocks given	1 (33.3%)
Follow-up status	
Lost to follow-up	4 (4.0%)
Death	1 (1.0%)
Withdrawal from follow-up	4 (4.0%)
Listed for LVAD or heart transplantation	1 (1.0%)
CRT lead-capping or extraction	1 (1.0%)
Surgical aortic valve replacement for severe aortic stenosis	1 (1.0%)
Percutaneous mechanical circulatory support device	1 (1.0%)

Proportions are presented as n (%). Data were available for all 101 patients unless otherwise specified. *CRT* Cardiac Resynchronization Therapy, *ICD* Implantable Cardioverter-Defibrillator, *LVAD* Left Ventricular Assist Device

For the whole cohort, electrical parameters of the systemic ventricular lead remained stable throughout follow-up. No significant differences were observed between baseline and 12-month follow-up in R-wave sensing (*p* = 0.327), capture threshold (*p* = 0.206), or lead impedance (*p* = 0.224), indicating consistent lead performance.

### Efficacy of CRT during 1-year follow-up

In total, 96 patients had at least one follow-up visit and 68 patients had a follow-up visit at 12 months (54 patients had a visit at 3 months, and 63 at 6 months). One patient died 13 months after the CRT implant due to end-stage HF, four patients were lost to follow-up, and four patients were withdrawn from follow-up. Of these, one patient was listed for heart transplantation, one underwent surgical aortic valve replacement for severe aortic stenosis, one received a percutaneous mechanical circulatory support device for acute hemodynamic support in the context of severe sepsis and septic shock, and in one patient CRT was discontinued due to coronary sinus lead dysfunction. As the indication for CRT was no longer present at that time, no new coronary sinus lead was implanted. In total, there were 10 HF events within the first year, involving 9 unique patients (8 urgent HF visits and 2 HF hospitalizations). At one year follow-up, 68 patients (72.3%) showed improvement of at least 1 class of NYHA and/or SVF.

#### QRS duration

**Fig. 1:**
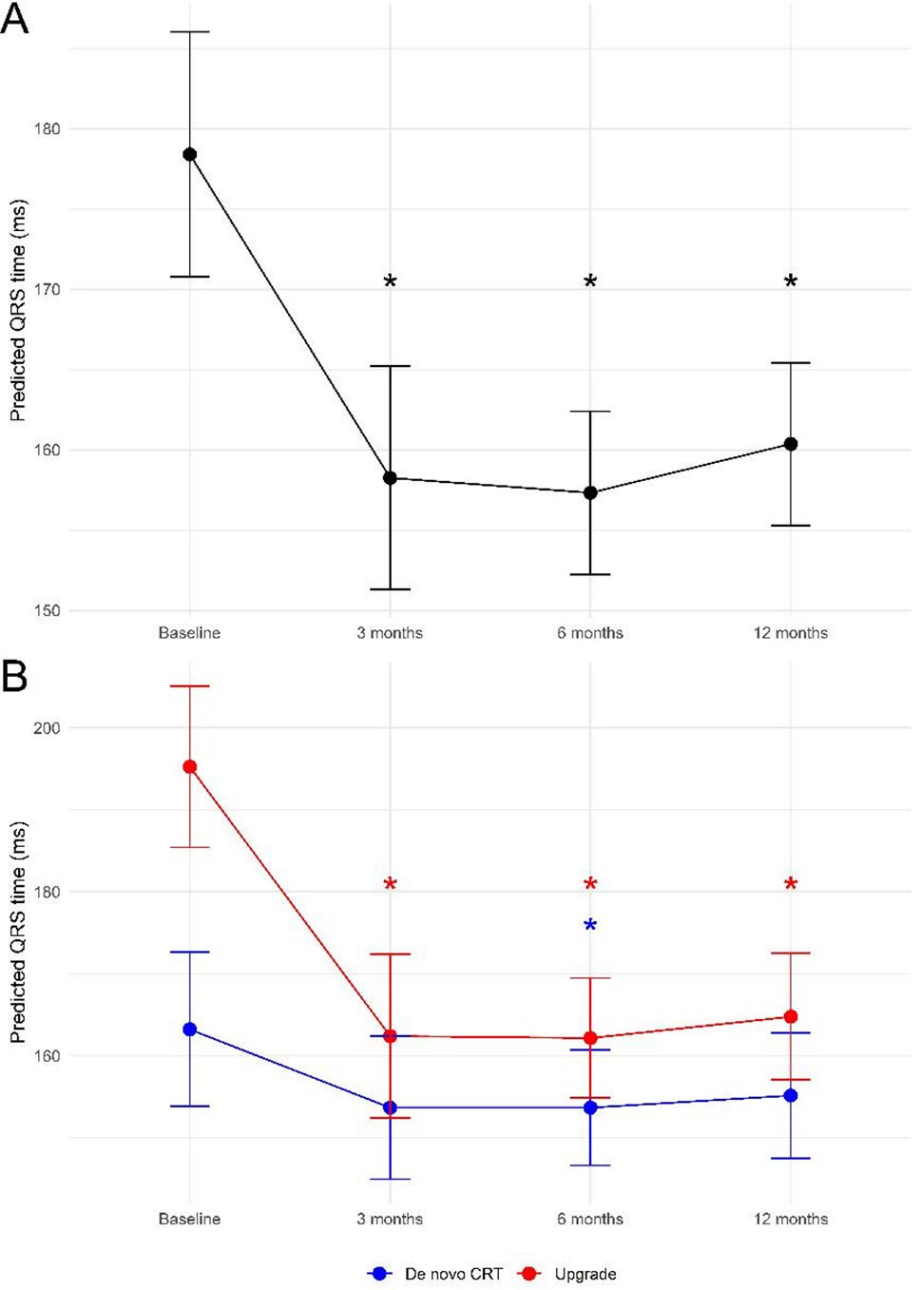
Change in QRS duration after CRT implantation. **A** shows the longitudinal changes in the model-estimated (predicted) QRS duration (ms) after CRT implantation in the whole population, based on the fixed effects of the linear mixed model. After implantation, there is a significant reduction in QRS duration across all time points. **B** shows that there is a differential effect between patients who received a de novo CRT implant and who underwent an upgrade procedure. At baseline, upgrade patients had a significantly longer QRS which reduced significantly at each time point. De novo CRT patients only had a significant QRS reduction at 6 months after implantation. The asterisks (*) indicate statistically significant changes

QRS duration reduced at 3, 6, and 12 months after CRT implantation (*p* < 0.001 for all time points) (Fig. 1A). At baseline, patients who underwent an upgrade to CRT had a significantly broader QRS than patients who underwent de novo CRT implantation (*p* < 0.001) (Fig. 1B). There was also a significant difference during follow-up between patients who underwent an upgrade procedure and patients who underwent de novo CRT implantation. Patients who underwent an upgrade procedure showed a reduction in QRS duration at each time point (*p* = 0.002 at 3, *p* < 0.001 at 6, and *p* < 0.001 at 12 months), while patients who underwent de novo CRT implantation had a less marked QRS narrowing and only demonstrated a significant QRS reduction at 6 months (*p* = 0.025). All exact mixed model results are presented in Table 4. The model characteristics are presented in Additional Table S1. The individual QRS changes are presented in Additional Figure S1, and the residual analyses for the mixed models are in Additional Figures S2 to S4.

**Table 4:** Mixed model outputs

	Estimate	95% CI	P-value
QRS model 1			
Baseline	178.40	170.76 – 186.03	<0.001
3 months vs baseline	-20.15	-27.18 – -13.11	<0.001
6 months vs baseline	-21.08	-27.49 – -14.67	<0.001
12 months vs baseline	-18.03	-24.68 – -11.39	<0.001
QRS model 2			
De novo CRT			
Baseline	163.21	153.81 – 172.62	0.000
3 months vs baseline	-9.55	-19.31 – 0.22	0.058
6 months vs baseline	-9.55	-17.82 – -1.28	0.025
12 months vs baseline	-8.06	-16.84 – 0.72	0.074
Upgrade CRT			
Baseline	195.24	181.65 – 208.84	0.000
3 months vs baseline	-32.84	-47.47 – -18.21	0.002
6 months vs baseline	-33.08	-44.96 – -21.20	<0.001
12 months vs baseline	-30.48	-42.99 – -17.96	<0.001

	OR	95% CI	P-value
SVF class model 1			
3 months vs baseline	0.21	0.05 – 0.90	0.035
6 months vs baseline	0.06	0.02 – 0.18	<0.001
12 months vs baseline	0.13	0.05 – 0.35	<0.001
SVF class model 2			
3 months vs baseline	0.21	0.05 – 0.88	0.032
6 months vs baseline	0.07	0.02 – 0.19	<0.001
12 months vs baseline	0.14	0.05 – 0.37	<0.001
LBBB at baseline	9.61	2.68 – 34.40	<0.001
sRV at baseline	12.00	2.13 – 67.60	0.005
NYHA class			
3 months vs baseline	0.11	0.03 – 0.40	<0.001
6 months vs baseline	0.04	0.01 – 0.17	<0.001
12 months vs baseline	0.06	0.02 – 0.21	<0.001

Estimates for the linear mixed models for continuous variable QRS duration in milliseconds are displayed as absolute baseline values and the change compared to baseline for the 3, 6, and 12 months follow-up intervals. Estimates for cumulative link mixed models for ordinal variable SVF class are displayed as the cumulative ORs, with an OR <1 constituting a lower odds of occupying a worse SVF class (= improvement) compared to baseline, and an OR >1 constituting a higher odds of occupying a worse SVF class. Estimates for generalized linear mixed models for binary variable NYHA class are displayed as ORs, with an OR <1 indicating a lower odds of having NYHA class >II (= improvement), and an OR >1 indicating a higher odds of having an NYHA class >II. All ORs were adjusted for intra‑patient clustering via random intercepts. *CI* Confidence interval, *CRT* Cardiac resynchronization therapy, *LBBB* Left bundle branch block, *NYHA* New York Heart Association, *OR* Odds ratio, *sRV* Systemic right ventricle, *SVF* Systemic ventricular function

#### SVF class

**Fig. 2:**
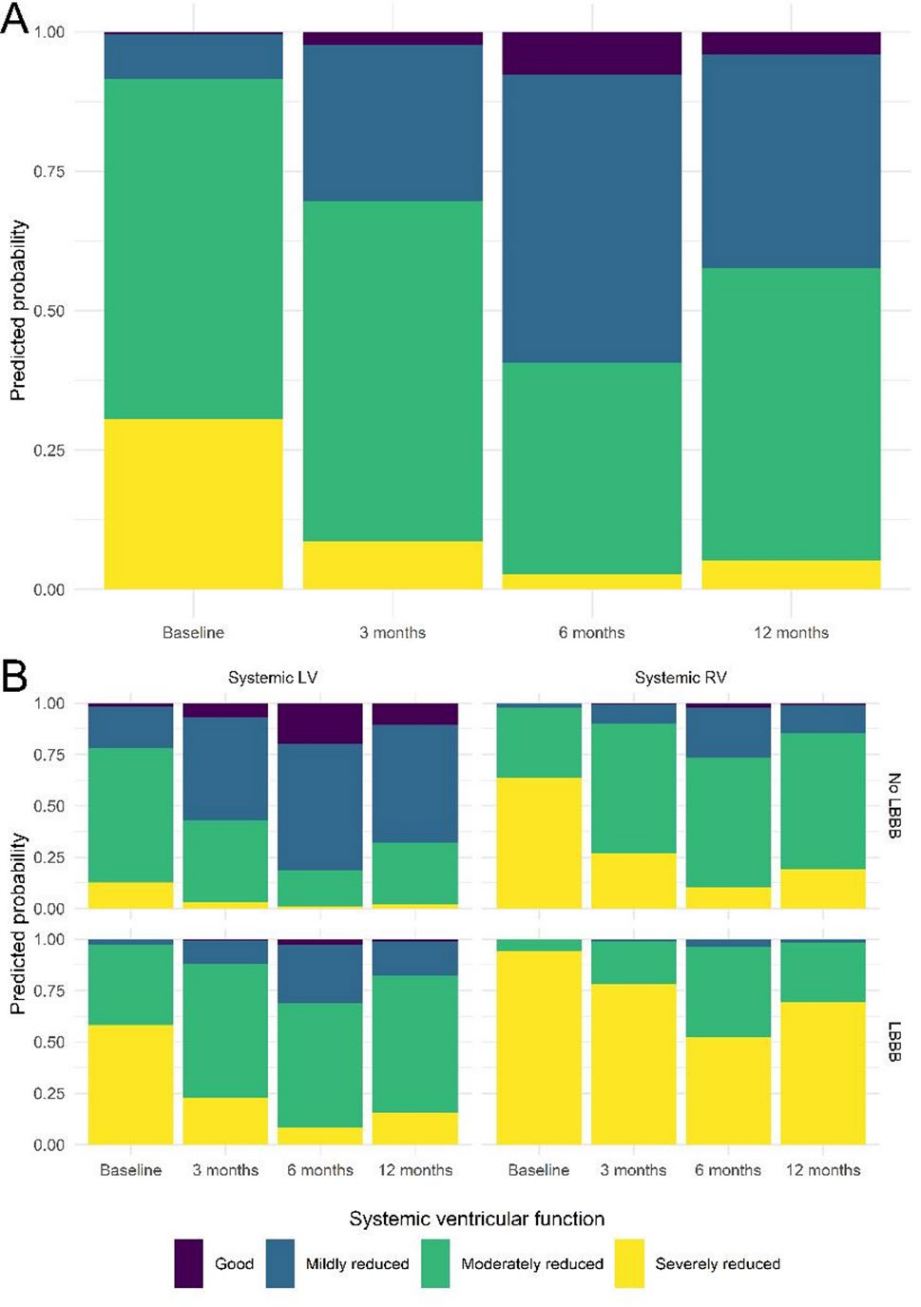
Change in SVF after CRT implantation. **A **the model-estimated (predicted) probabilities for each class of systemic ventricular function are presented, calculated from the fixed effects of the cumulative link mixed model. There is a significant improvement in SVF at each follow-up moment. **B **the presence of an LBBB at baseline and a sRV is associated with significantly worse overall SVF, but the longitudinal improvements remain significant in all groups. *CRT* Cardiac resynchronization therapy, *LBBB* Left bundle branch block, *LV* Left ventricle, *(s)RV* (systemic) right ventricle, *SVF* Systemic ventricular function

SVF class improved significantly at 3 (*p* = 0.035), 6 (*p* < 0.001), and 12 months (*p* < 0.001) after CRT implantation, as can be appreciated from Fig. 2A. This improvement remained significant across all time points, regardless of the presence of LBBB at baseline or systemic ventricle anatomy (Fig. 2B). Nevertheless, patients with a LBBB at baseline had significantly higher cumulative odds for worse overall SVF than patients without LBBB (*p* < 0.001). Similarly, sRV patients had significantly higher cumulative odds for worse overall SVF than sLV patients (*p* = 0.005).

#### NYHA class

**Fig. 3:**
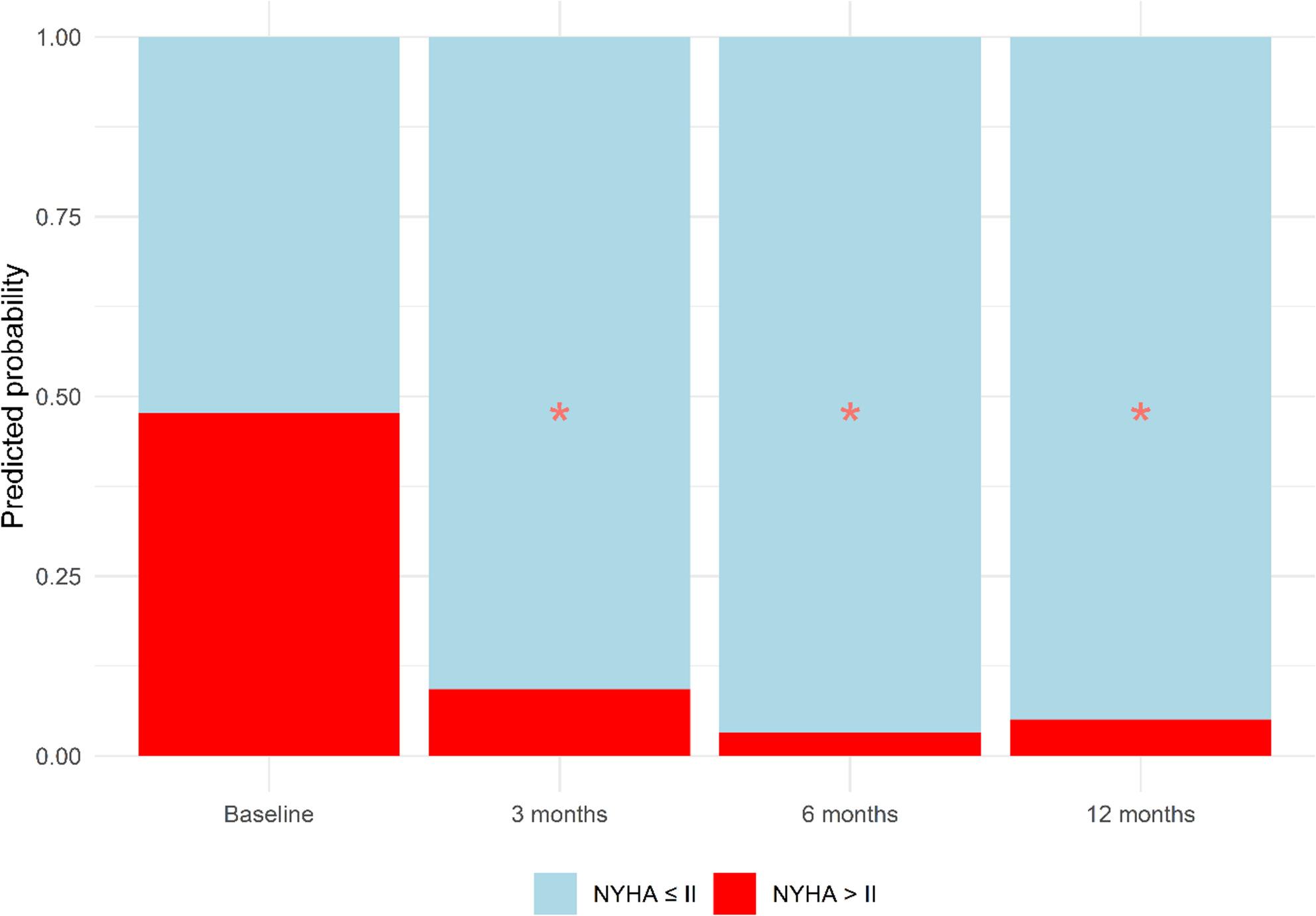
Change in NYHA class after CRT implantation. Model-estimated (predicted) probabilities of having NYHA class ≤ II (light blue) or > II (red) at each follow-up moment are presented, derived from the fixed effects of the generalized linear mixed model. At baseline, there is an even distribution between groups. After CRT implantation, there is a significant improvement in NYHA class, with a reduction in the probability of having NYHA class > II at each follow-up moment. The asterisks (*) indicate statistically significant changes. *CRT* Cardiac resynchronization therapy, *NYHA* New York Heart Association

NYHA class improved significantly at 3, 6, and 12 months after CRT implantation (*p* < 0.001 for all time points) (Fig. 3). None of the covariates (de novo CRT versus upgrade, LBBB versus non-LBBB at baseline, or sLV versus sRV) significantly influenced the observed improvements in NYHA class after CRT. A summary of the main findings is presented in the Graphical Abstract.

## Discussion

In this real-world cohort of 101 ACHD patients, a significant reduction in QRS duration, improvement in SVF, and improvement in NYHA functional class were observed in the first year after CRT implantation. These benefits were observed across the spectrum of ACHD complexity and extended to patients with a sRV and non-LBBB QRS morphology. Patients undergoing an upgrade from a pre-existing pacing system experienced a more pronounced reduction in QRS duration compared to those receiving a de novo implant. The overall complication rate was low, underscoring the safety and feasibility of CRT in this anatomically complex and clinically heterogeneous population.

### Impact on QRS duration

CRT implantation was associated with a significant reduction in QRS duration, particularly among patients undergoing upgrade from a pre-existing CIED. A likely explanation for this finding is that patients with a pre-existing device often had a paced baseline QRS complex, which could account for the more substantial reduction in QRS duration after CRT implantation by mitigating the subpulmonary pacing-induced QRS broadening and dyssynchrony. This difference between upgrade and de novo patients is in line with the previous observations of Dubin et al. [16]. However, the clinical significance of QRS narrowing as a surrogate for CRT response remains uncertain in this population. Yin et al. identified baseline QRS duration as the only significant independent predictor of CRT response in a cohort of 70 ACHD patients [8]. In contrast, Thompson et al. found no significant difference in baseline QRS duration between responders and non-responders, and paradoxically, post-procedure QRS duration was even shorter in non-responders than in responders, with response defined as a ≥ 1-class improvement in NYHA and/or SVF [10]. These seemingly contradictory findings may be explained by the complex nature of electromechanical coupling in ACHD, where improvements in electrical synchrony on the intra- and interventricular level may not necessarily translate into mechanical synchrony [10].

### Impact of QRS morphology on the response to CRT

In patients with structurally normal hearts, LBBB morphology predicts better CRT response compared to non-LBBB [17]. In our cohort, however, 66.3% had non-LBBB QRS morphology at baseline. Bundle branch block, especially RBBB, is more complex in ACHD and should not be perceived in an identical manner to non-congenital populations. RBBB in ACHD can be the result of surgical incision and scarring, but also from hemodynamic (volume and/or pressure) overload of the RV, either impacting the RV or directly damaging the conduction system [18]. It is therefore not surprising that Yin et al. showed that stratification according to conventional QRS morphology criteria was not related to echocardiographic response to CRT in ACHD patients [8]. Our findings support this conclusion, as patients with a non-LBBB pattern on baseline ECG demonstrate similar improvements in QRS duration, SVF class, and NYHA functional class compared to those with a LBBB morphology at baseline. Current guidelines for patients with structurally normal hearts and non-LBBB may thus not be directly applicable to the ACHD population. This is also reflected by the significant proportion of patients in our cohort (*n* = 15, 14.9%) who received CRT, despite not meeting conventional guideline-based indications, where the decision was made on expert consensus. In the vast majority of these cases (*n* = 14, 93.3%), the indication was a (perceived) high pacing requirement in the context of a good or only mildly reduced SVF. The expert panel opted for early CRT implantation rather than waiting for further ventricular function decline. Notably, our analysis showed improvement in SVF across all baseline SVF classes, suggesting that this approach may be beneficial, although it warrants further investigation.

### Impact of systemic ventricular morphology on the response to CRT

Experience with CRT in patients with a sRV is limited, and results are variable. Some studies reported less favorable results, especially when compared to CRT in patients with sLV [8, 19–21], while others report favorable outcomes [16, 22]. One of the largest retrospective studies to date included 80 patients with sRV and showed a significant improvement in functional class and QRS duration only after upgrade to CRT, and not after a de novo implantation [23]. Nonetheless, a recent retrospective multicenter study including 105 adult sRV patients undergoing CRT found no improvement in overall survival and reported that CRT was, in fact, associated with worse survival compared with matched controls [24]. Although the authors used propensity-score matching to adjust for baseline differences, this association may still reflect residual confounding, as in current practice, CRT is more likely to be offered to higher-risk patients with more advanced disease. In our cohort, we observed a significant reduction in QRS duration in patients with sRV following CRT, as well as an improvement in NYHA functional class and SVF. While sRV patients had worse overall SVF compared to sLV patients, and patients with a combination of an sRV and LBBB morphology exhibited the most advanced SVF impairment, the observed longitudinal improvements were statistically significant across all groups. These findings suggest that CRT should not be dismissed based on systemic ventricular anatomy and/or LBBB morphology. Due to the relatively low number of sRV patients in the current cohort and the lack of hard clinical endpoints, no definitive conclusions can be drawn about whether there is a difference in response between sRV patients undergoing upgrade or de novo CRT implantation, nor between ccTGA patients and TGA patients after Mustard. Interestingly, no patients with a single ventricle physiology who received CRT with multi-site ventricular pacing were identified at our centers, likely reflecting the anatomical and physiological complexity inherent to single ventricle anatomies, although early findings seem encouraging [25].

### Adverse events

The early implant-related complication rate in our cohort was relatively low, with only 6 patients (5.9%) requiring re-intervention within the first year. This is lower than the 11% reported in the recent retrospective cohort study by Yin et al., although their mean follow-up of 4.7 years was considerably longer [8]. Earlier studies documented adverse event rates as high as 29% [16]. This discrepancy likely reflects advancements in device technology and procedural techniques over the past decades rather than differences in operator performance. Given the low complication rates with contemporary technology and the availability of a wide range of catheters, leads, and implantation techniques, CRT for ACHD appears to be a relatively safe procedure. Considering the heterogeneous anatomy in this population, referral to experienced centers may be advisable, though ACHD should not be seen as a contraindication per se for CRT implantation by skilled operators. Conduction system pacing may offer a promising alternative in selected ACHD patients, particularly when conventional CRT delivery is challenging. While early data on His bundle pacing and left bundle branch area pacing are encouraging, there is limited available data and their broader applicability in the anatomically diverse ACHD population remains uncertain and warrants further prospective evaluation [26–29].

### Study limitations

First, this study is inherently limited by the retrospective, single-arm design with limited follow-up duration. Lack of a control group that did not receive CRT prevents us from making direct comparisons, and lack of data on hard endpoints limits the ability to draw conclusions regarding the long-term benefit of CRT in the course of ACHD-related HF. Second, selection bias is inherent, as some ACHD patients with an indication for CRT may not have undergone the procedure due to various factors, and patients in whom CRT implantation was initially deemed unfeasible or unsuccessful were not captured in this study. Third, the indications for CRT were determined on a case-by-case basis, leading to a heterogeneous cohort with a range of congenital defects and variable follow-up protocols. While this introduces variability, it also reflects the real-world complexity inherent to the management of ACHD patients with HF. Fourth, assessing ventricular function in ACHD patients can be challenging, and there may be intra- and interobserver variability in these measurements. To address this, we categorized SVF based on a classification system (normal, mildly, moderately, or severely reduced) to minimize discrepancies. Fifth, follow-up data were incomplete for some participants. Mixed models were used to account for this unbalanced data by incorporating all available data and the within-patient correlation of repeated measurements, minimizing bias compared with conventional paired analyses.

## Conclusions

In summary, this real-world, multicenter study of 101 ACHD patients undergoing CRT implantation demonstrates significant improvements in QRS duration, systolic SVF, and NYHA functional class in the first year following implantation. Importantly, these benefits were also observed in patients with a sRV and those with a non-LBBB QRS morphology—two patient groups outside the traditional established guidelines. Patients undergoing device upgrades experienced a more pronounced reduction in QRS duration than those receiving de novo implants, but SVF and NYHA improved significantly in both subgroups. The overall complication rate was low. These findings provide further evidence supporting the use of CRT as a safe, feasible, and effective therapy for ACHD patients with HF. ACHD patients should not be disregarded as candidates for CRT based on their intricate anatomy and ventricular morphology, and indications might extend beyond the established guidelines for patients without congenital heart disease.

## Supplementary Information

Supplementary Material 1.

## Data Availability

The datasets used and/or analysed during the current study are available from the corresponding author on reasonable request.

## Abbreviations

ACHD: Adult congenital heart disease
(cc)TGA: (Congenitally corrected) transposition of the great arteries
CIED: Cardiac implantable electronic device
CRT: Cardiac resynchronization therapy
ECG: Electrocardiogram
HF: Heart failure
LBBB: Left bundle branch block
NYHA: New York Heart Association
RBBB: Right bundle branch block
Slv: Systemic left ventricle
sRV: Systemic right ventricle
SVF: Systemic ventricular function
