# Supplementary material for: Effects of cardiac resynchronization therapy in adults with congenital heart disease
Source: BMC Cardiovasc Disord. 2026 Apr 7;26:423. doi: 10.1186/s12872-026-05818-5 (PMC13195909; doi:10.1186/s12872-026-05818-5)
Supplement: Supplementary file 1 — Supplementary Material 1. [file 12872_2026_5818_MOESM1_ESM.docx]

**ADDITIONAL FILES**

**Title:** Real-World Data on the Effects of Cardiac Resynchronization Therapy in Adults with Congenital Heart Disease

**Contents**

[**ADDITIONAL TABLES** 2](#_Toc202863146)

[**Additional Table S1** 2](#_Toc202863147)

[**ADDITIONAL FIGURES** 3](#_Toc202863148)

[**Additional Figure S1** 3](#_Toc202863149)

[**Additional Figure S2** 4](#_Toc202863150)

[**Additional Figure S3** 6](#_Toc202863151)

[**Additional Figure S4** 8](#_Toc202863152)

# **ADDITIONAL TABLES**

## **Additional Table S1**

Mixed model characteristics

|  | **Mixed model structure** | **Variance structure** | **Significant covariates** |
| --- | --- | --- | --- |
| QRS model 1 | Continuous variable. Linear mixed model with random intercepts and random slopes | Unspecified | Not included |
| QRS model 2 | Continuous variable. Linear mixed model with random intercepts | VarIdent for time variable | De novo versus upgrade CRT procedure |
| SVF class model 1 | Ordinal variable. Cumulative link mixed model. | N/A | Not included |
| SVF class model 2 | Ordinal variable. Cumulative link mixed model. | N/A | LBBB at baseline & systemic ventricle anatomy as fixed effects. No longitudinal effects |
| NYHA class | Binary variable. Generalized linear mixed model with random intercepts | N/A | No significant covariates |
| *CRT, cardiac resynchronization therapy; LBBB, left bundle branch block; NYHA, New York Heart Association; SVF, systemic ventricular function.* | | | |

# **ADDITIONAL FIGURES**

## **Additional Figure S1**

Individual changes in QRS duration

**
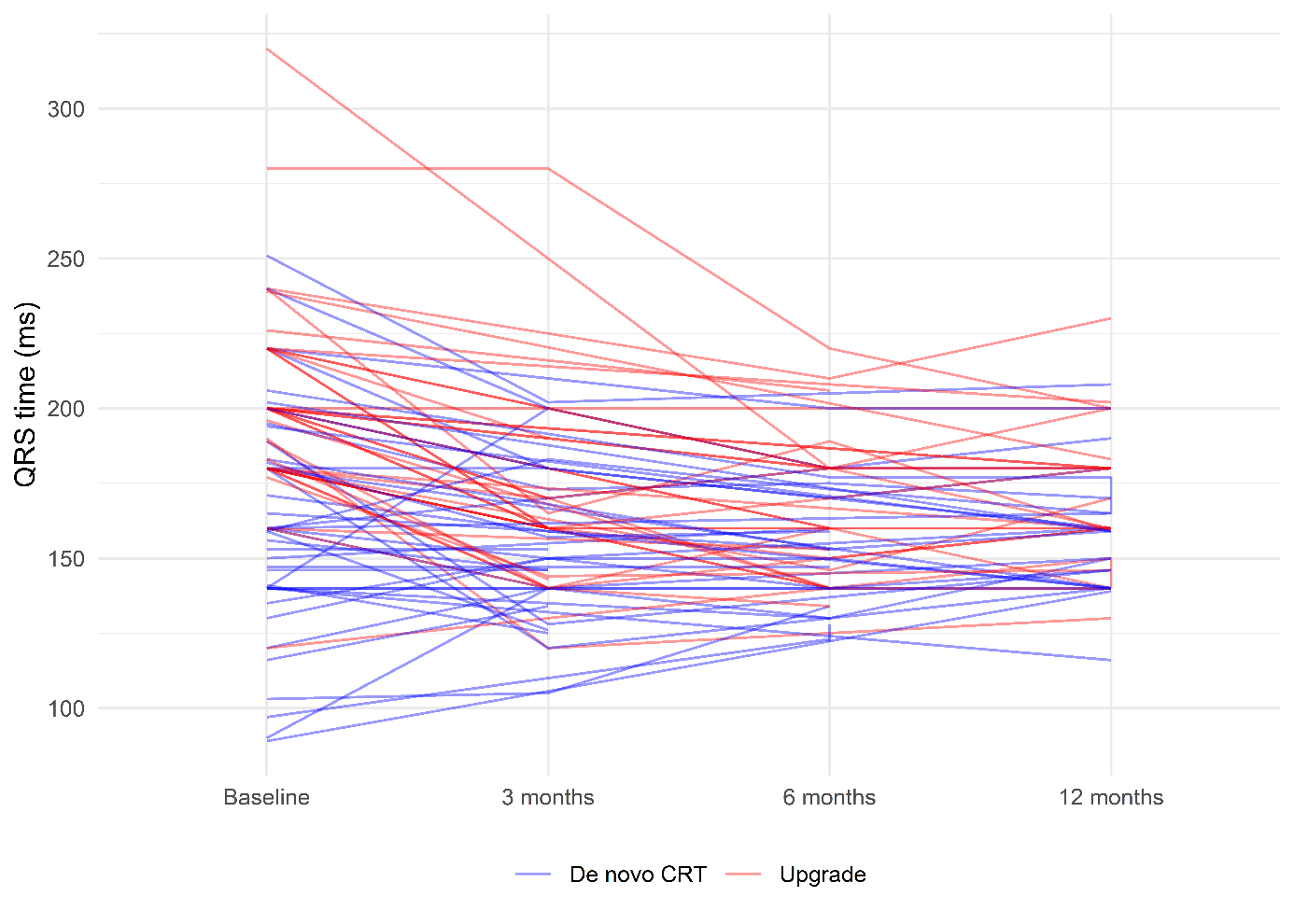
**

**Additional Figure S1 Legend.** Spaghetti plot showing the individual changes in QRS duration after CRT implantation, separated for patients who underwent de novo CRT implant (blue) and patients who underwent an upgrade procedure (red). *CRT, cardiac resynchronization therapy.*

## **Additional Figure S2**

QRS models diagnostics

A)


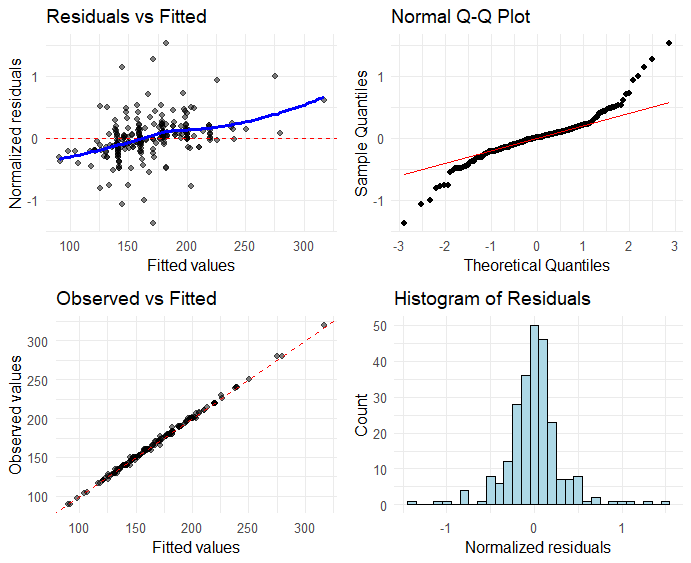


B)


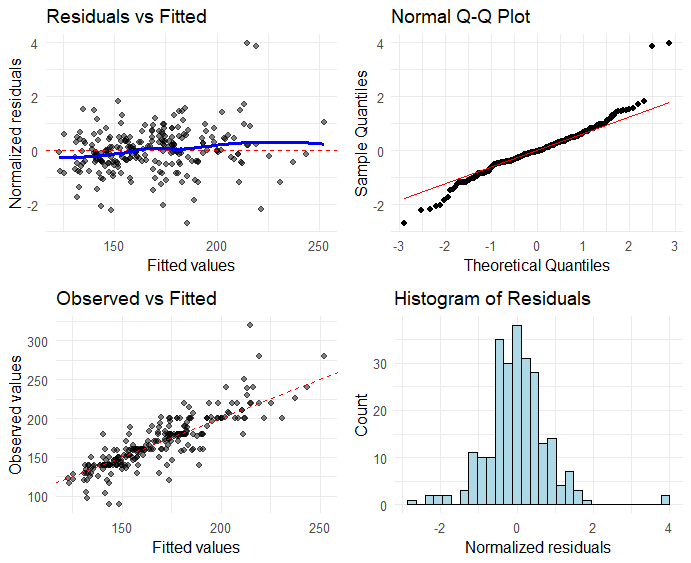


**Additional Figure S2 Legend.** A) shows 4 residuals plots for the QRS model without covariates. There is a subtle increasing trend in the normalized residuals with higher fitted values. There is limited departure from normality. The fitted values closely follow the observed values. These plots suggests a good model fit. B) shows 4 residuals plots for the QRS model with the significant covariate de novo vs upgrade CRT procedure. There is less of an increase with higher fitted values, no major deviation from normality and good correlation between fitted and observed values, again suggesting good model fit. *CRT, cardiac resynchronization therapy.*

## **Additional Figure S3**

Systemic ventricular function class models diagnostics

A)


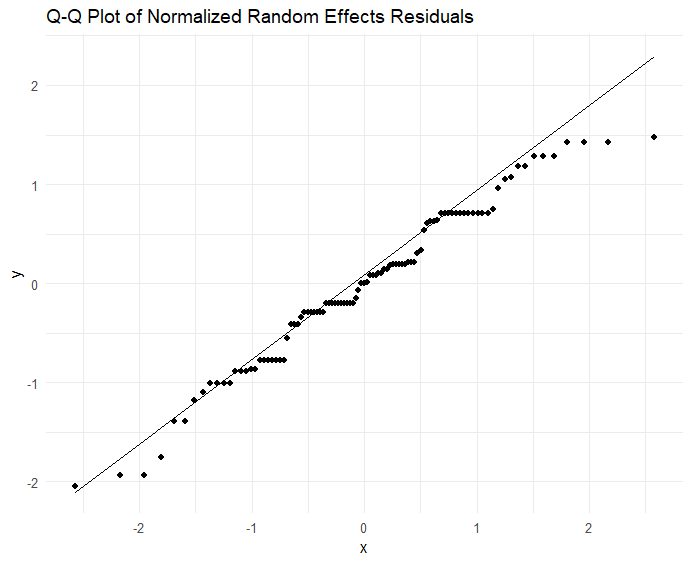


B)


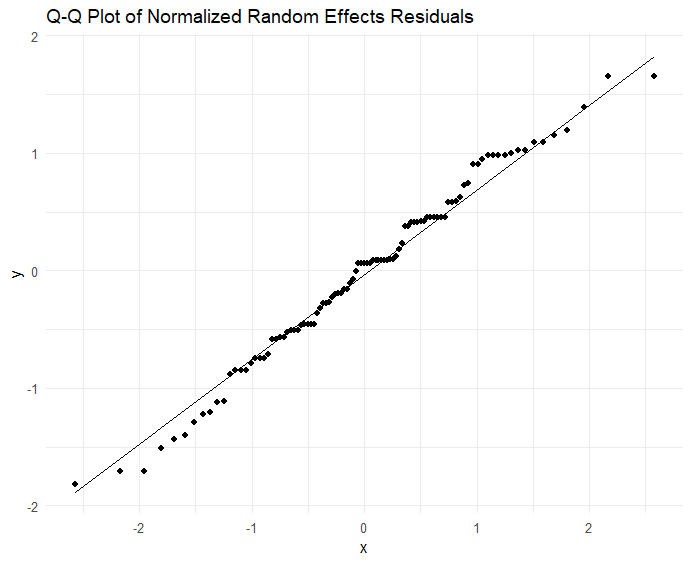


**Additional Figure S3 Legend.** Conventional residuals analyses are not suitable for cumulative link mixed models. The proportional odds assumption was tested and it was not violated. Panel A shows the distribution of the normalized random effects residuals is normal, suggesting appropriate model fit. Panel B shows the same plot but for the model with the significant fixed effect covariates LBBB at baseline and systemic ventricle anatomy. The model with the covariates included seems to fit the normality assumption slightly better. *LBBB, left bundle branch block.*

## **Additional Figure S4**

NYHA class model diagnostics


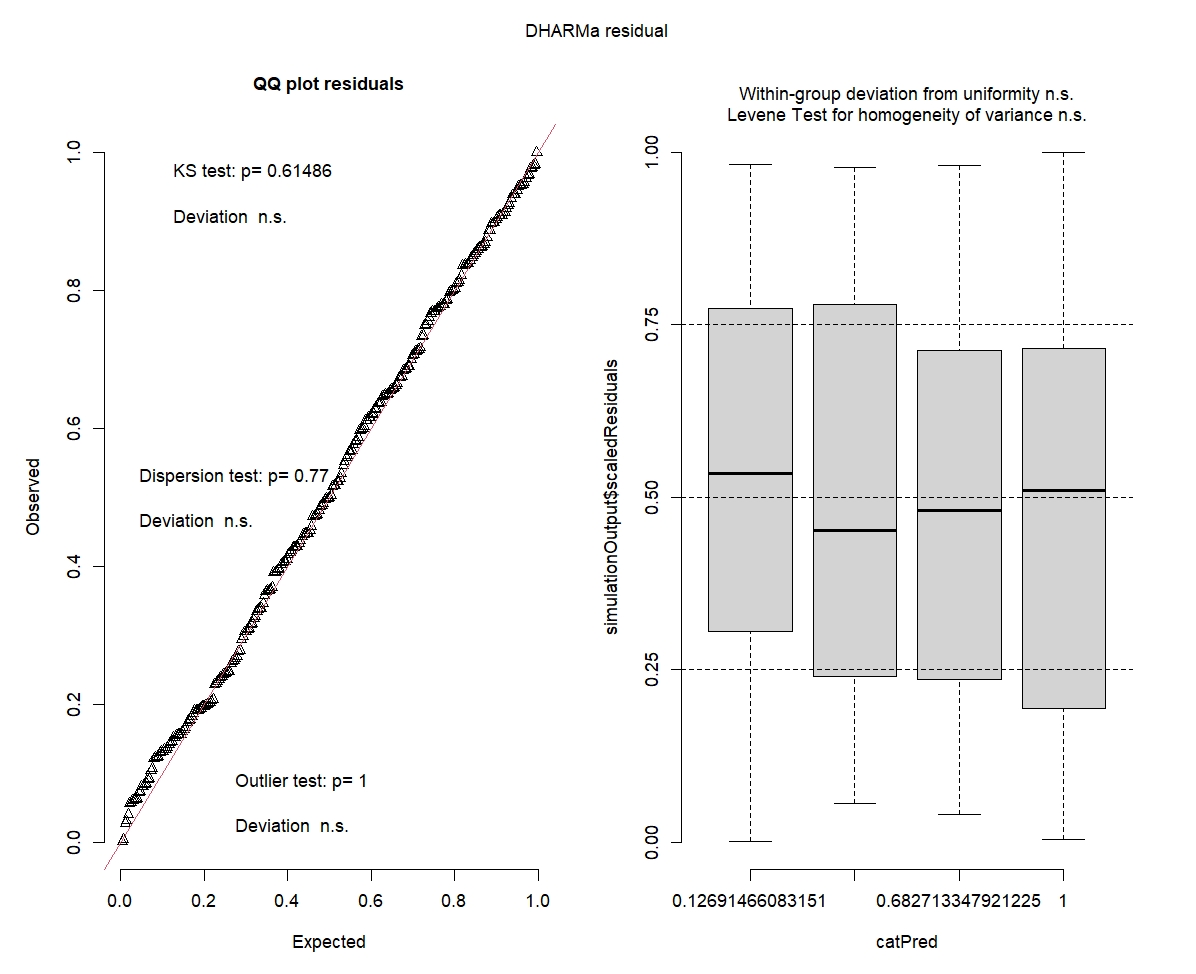


**Additional Figure S4 Legend.** The first panel shows a QQ plot with all points following the reference line closely, indicating that the residuals are distributed well. The Kolmogorov-Smirnov (KS) test is non-significant (p=0.6148). This suggests no significant variation from the expected distribution. The dispersion test and outlier test are also non-significant (p=0.77 and p=1 respectively), indicating no evidence of over- or under-dispersion nor significant outliers. The second panel shows the residuals versus predicted values. There are no signs of heteroskedasticity and the residuals appear uniformly distributed within each group of predicted values. *KS, Kolmogorov-Smirnov; NYHA, New York Heart Association.*
